# Reviewing the evidence on effectiveness and cost-effectiveness of HIV prevention strategies in Thailand

**DOI:** 10.1186/1471-2458-10-401

**Published:** 2010-07-07

**Authors:** Juntana Pattanaphesaj, Yot Teerawattananon

**Affiliations:** 1 Health Intervention and Technology Assessment Program (HITAP), Ministry of Public Health, Tiwanon Road, Muang, Nonthaburi, 11000, Thailand

## Abstract

**Background:**

Following universal access to antiretroviral therapy in Thailand, evidence from National AIDS Spending Assessment indicates a decreasing proportion of expenditure on prevention interventions. To prompt policymakers to revitalize HIV prevention, this study identifies a comprehensive list of HIV/AIDs preventive interventions that are likely to be effective and cost-effective in Thailand.

**Methods:**

A systematic review of the national and international literature on HIV prevention strategies from 1997 to 2008 was undertaken. The outcomes used to consider the effectiveness of HIV prevention interventions were changes in HIV risk behaviour and HIV incidence. Economic evaluations that presented their results in terms of cost per HIV infection averted or cost per quality-adjusted life year (QALY) gained were also included. All studies were assessed against quality criteria.

**Results:**

The findings demonstrated that school based-sex education plus life-skill programs, voluntary and routine HIV counselling and testing, male condoms, street outreach programs, needle and syringe programs, programs for the prevention of mother-to-child HIV transmission, male circumcision, screening blood products and donated organs for HIV, and increased alcohol tax were all effective in reducing HIV infection among target populations in a cost-effective manner.

**Conclusion:**

We found very limited local evidence regarding the effectiveness of HIV interventions amongst specific high risk populations. This underlines the urgent need to prioritise health research resources to assess the effectiveness and cost-effectiveness of HIV interventions aimed at reducing HIV infection among high risk groups in Thailand.

## Background

Since the introduction of the universal health insurance coverage policy in 2001, Thailand has sought to further ensure efficient resource allocation in the health sector [1]. Evidence-based decision making requires that decisions about health and health care are based on best available information. To use such an approach it is necessary to appraise what constitutes evidence in relation to health-enhancing interventions. While the use of effectiveness information alone to justify health care resource allocation is still common practice, decision makers, academics and health care professionals are increasingly interested in cost-effectiveness data to guide policy making. Such evaluation are designed to guide explicit resource allocation decisions by comparing the incremental costs and consequences of alternative health care interventions [2].

Thailand is classified as facing a mixture of concentrated and generalized epidemics of HIV infection[3], similar to other countries such as South Africa, Egypt, Russia, and Papua New Guinea [4]. High HIV prevalence has been observed among some particular populations, for example, men who have sex with men, injecting drug users (IDUs), and female sex workers, while HIV prevalence among pregnant women was relatively low (0.8%) [5]. It is noteworthy that the prioritisation of strategies for dealing with sexually transmitted infections and HIV/AIDS was a product of their perceived high disease burden, although often without reliable evidence regarding the effectiveness and cost-effectiveness of the interventions themselves. Furthermore, many HIV/AIDS programs have been implemented without close monitoring, or rarely incorporated well-defined control or comparison groups necessary to identify their actual effect size. In parallel to these prevention strategies, a policy for universal antiretroviral treatment has been in operation in Thailand since 2001, with almost 150,000 individuals living with HIV/AIDS currently reaching the program. Evidence from the National AIDS Spending Assessment indicated a decreasing proportion of expenditure on prevention intervention from 18% in 2000 to 13% in 2004 [6].

Although there were some prior attempts to provide information for guiding policy decisions regarding resource allocation towards HIV prevention, these all had limitations in their applicability to the Thai health care setting. Bollinger [7] provides an extensive review of the literature assessing impacts of HIV prevention interventions in developing settings and applied a matrix of effectiveness coefficients to prioritize the interventions. His major finding is that interventions that included interpersonal contact offered a greater impact than interventions targeting a more general audience; however, the impact was measured only in terms of sexual behaviour change and did not include program costs as a prioritizated criterion. Meanwhile, Cohen et al [8] and the second edition of "Disease Control Priorities in Developing Countries" (hereafter "DCP2") [9] adopted a 'maximization concept' by using economic evaluation as the primary resource allocation criterion but neither of them was considered applicable to the Thai setting; Cohen et al provide an HIV prevention cost-effectiveness league table tailored to the US setting while DCP2 provided policy recommendations on HIV prevention across a broader range of health care settings.

This paper addresses the need to assess the usefulness and value for money of HIV/AIDS prevention interventions. It aims to make a comprehensive list of interventions that are likely to be effective and cost-effective in the Thai setting. This information can be crucial for guiding public investment to lessen both the short and long-term impacts of HIV/AIDS in Thailand by re-emphasising the role of prevention as compared with that of treatment in managing the epidemic.

## Methods

### Scope and type of interventions

Interventions included in this review were those that showed evidence of reducing HIV incidence or risk behaviours likely to effect horizontal and vertical HIV transmission. The set of interventions was not restricted to those used in practice in Thailand or funded by the Thai government. It also covered interventions provided at all levels, i.e. individuals, groups, and communities which are likely to be beneficial in the reduction of the HIV/AIDS epidemic worldwide.

Given that a wide range of interventions were included in this study, it is imperative that they have clear definitions and detailed information to ensure a better understanding of, for example, their precise aims, what their delivery modes are, and at whom the interventions are targeted. A lack of clarity and descriptive detail of interventions makes it difficult to assess and compare either the effectiveness or cost-effectiveness in different settings. It is also impossible to make sensible recommendations with regards to policy decision making if there are no concise definitions for commonly implemented intervention approaches.

It was necessary, therefore, for this study to establish or adopt a standard structure to define and classify interventions for the prevention of HIV/AIDS. Based on the recommendations made by UNAIDS [10], HIV prevention interventions are grouped into five broad categories as follows:

1. Interventions that affect knowledge, attitude and beliefs and influence psychological and social correlates of risk;

2. Harm reduction interventions that lower the risk of a behaviour, but do not eliminate the behaviour;

3. Biological/biomedical interventions that strive to reduce HIV infection and transmission risk;

4. Mitigation of barriers to prevention and negative social outcomes of HIV infection;

5. Mitigation of biological outcomes of HIV infection.

However, as the fifth category was not related to HIV prevention, it was not included in this review. From the above recommendations, we provide a definition and classification of each HIV prevention intervention in additional file 1: Table S1.

### Sources of information

We obtained literature published in Thai or English from 1997 to 2008. The studies conducted within a Thai setting were given a higher priority since they better recognise the resource and infrastructure limitations that are specific to the health care system in Thailand, as well as the effectiveness of interventions which are affected by context specific factors. The review of the Thai literature, therefore, included both published and unpublished (grey) literature such as research reports, Master's dissertations or Ph.D. theses, which are considered highly relevant to the Thai context. If local data regarding effectiveness or cost-effectiveness of an intervention were not available, then a systematic search of Pubmed and Cochrane databases was carried out. A broad search strategy with appropriate MeSH terms and keywords was used in combination with a filter to restrict type of studies to systematic reviews, meta-analysis, randomized controlled trial, case control studies or cohort studies. Search terms used included 'acquired immunodeficiency syndrome', 'HIV', 'prevention and control', 'primary prevention', 'intervention studies', or 'early intervention'. Exclusion criteria were: i) publications of the same study, ii) descriptive studies, iii) assessment of satisfaction, knowledge and attitude towards HIV/AIDS, risk behaviours and program activities (not outcomes), iv) reports of case studies, and v) cost analyses.

### Effectiveness of interventions

**Figure 1:**
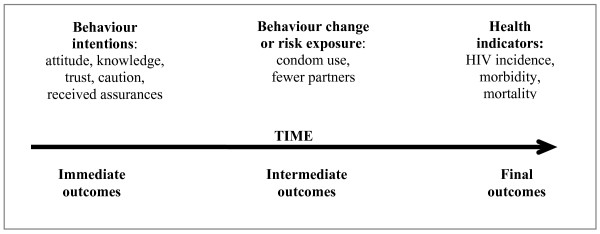
**Outcome measures for HIV prevention interventions**.

The effectiveness of interventions can be measured in a number of ways. Figure 1 (adapted from [11]) shows the concept of outcome hierarchies that emphasize the difference between 'immediate', 'intermediate' and 'final' outcomes of HIV interventions. Immediate measures of effectiveness of HIV interventions are characterised by change in knowledge, attitude, perception and skills of the individuals. In many HIV programs, the changes were reported in terms of trust, caution and received assurances. Further along the continuum, these immediate changes can subsequently affect health behaviours and health determinants, for example, condom use, abstinence, sharing injection equipments, or fewer sexual partners. Finally, changes in incidence or morbidity or mortality should be evaluated as the final or ultimate goal of the program. The primary criterion for selection of studies was that they report the effectiveness in terms of the changes in HIV risk behaviour and HIV incidence. We deliberately excluded descriptive or qualitative reports from the review as these most often do not provide comparable measures of outcome. Studies reporting outcomes in terms of improvement in attitude or knowledge were also excluded because changes in these are not always directly associated with behavior change [12].

### Cost-effectiveness of interventions

The review included economic evaluation studies that presented their results in terms of cost per HIV infection averted, or cost per quality-adjusted life year (QALY) gained, or cost per disability-adjusted life year (DALY) averted. This review compared the value for money of different HIV/AIDS preventive interventions using a societal viewpoint since it is most relevant to priority setting in health care. However, if the evaluation took the more limited health care provider perspective, this was used instead.

Differences in monetary currencies and unit costs between locations and over time are among the most commonly cited obstacles to applying economic evaluation findings across different settings. This study adjusted all cost-effectiveness ratios in a common currency, the international dollar, at their 2008 value, using national consumer price index (CPI) and purchasing power parity (PPP) information from the International Monetary Fund (IMF) [13].

With regard to the thresholds for considering an intervention to be cost-effective, the Subcommittee for development of the National List of Essential Medicines in Thailand developed its own threshold as a criterion to include or exclude medicines from the reimbursement list [14]. Interventions with a cost per QALY gained below 100,000 THB (approximating the Thai GDP per capita) are considered favourably for inclusion. Given the fact that 11.23 QALYs could be saved from one HIV infection avoided [15], the thresholds for one HIV infection averted was approximately 90,000 PPP$.

### Quality assessment

Systematic reviews and meta-analyses of high quality RCTs were considered the most favourable data sources [2]. The advantages of using systematic reviews of clinical effects are twofold. First, a more precise estimate can be attained from combining the outcome data from a number of studies. Second, by using the results from studies carried out in a range of settings, assuming that these studies are sufficiently homogenous to be comparable, the estimate can then be applied to a more general patient population with different baseline risks, rather than specifically for a population group selected for an individual trial. In cases where a meta-analysis of RCT(s) was not available alternative evidence sources were used in accordance with the hierarchy shown in table 1.

**Table 1:** Levels of clinical evidence

A+	Systematic reviews & meta-analyses of RCTs or RCT(s) conducted in Thailand with a very low risk of bias*.
A	Systematic reviews & meta-analyses of RCTs or RCT(s) conducted internationally with a very low risk of bias.

B+	Systematic reviews & meta-analyses of RCTs or RCT(s) conducted in Thailand with a high risk of bias.

B	Systematic reviews & meta-analyses of RCTs or RCT(s) conducted internationally with a high risk of bias.

C+	Systematic reviews of case control or cohort studies conducted in Thailand with a very low risk of confounding, bias, or chance and a high probability that the relationship is causal.

C	Systematic reviews of case control or cohort studies conducted internationally with a very low risk of confounding, bias, or chance and a high probability that the relationship is causal.

D+	Case control or cohort studies conducted in Thailand with a high risk of confounding, bias, or chance and a significant risk that the relationship is not causal.

D	Case control or cohort studies conducted internationally with a high risk of confounding, bias, or chance and a significant risk that the relationship is not causal.

* A bias is a systematic error, or deviation from the truth, in results or inferences.

## Results

### Description of studies

**Figure 2:**
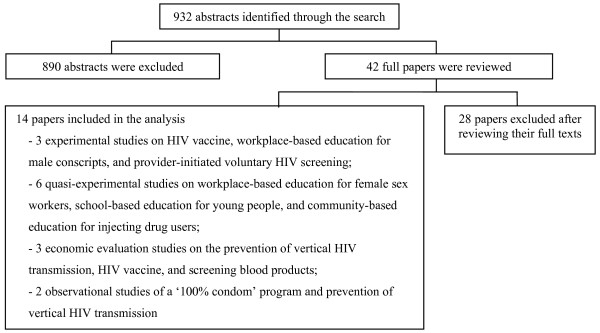
**Literature review profile of the Thai literature**.

Searching the Thai databases initially identified a total of 932 abstracts (see figure 2). Of these, 890 abstracts were excluded based on our exclusion criteria. From the full text review of the remaining 42 papers, only fourteen papers were found to be relevant and included in the analysis. Of the 28 papers excluded, 25 papers reported only immediate outcomes of the HIV prevention programs. For example, two papers that reported the effectiveness of the distribution of condom vending machines in the communities, used only numbers of condoms sold per machine and/or customer's satisfaction as their outcome measures [16,17]. Three other papers that evaluated drug regimens for the prevention of vertical HIV transmission, were excluded because the regimen under investigation(AZT regimens), is no longer used in clinical practice in Thailand [18-20]. The remaining 14 studies [21-34] were included in the analysis.

**Figure 3:**
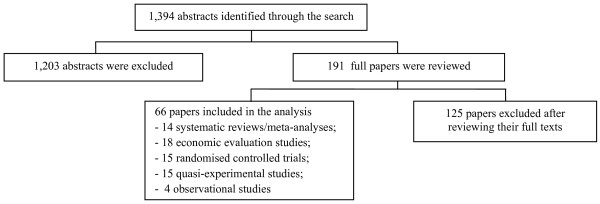
**Literature review profile of the international literature**.

We identified 1,394 abstracts from the international literature (see figure 3). After reading the abstracts, 1,203 studies were eliminated because they were editorials, descriptive, or qualitative reports. In addition, we also excluded a number of studies that assessed the effectiveness and cost-effectiveness of programs for the prevention of mother-to-child HIV transmission because relevant Thai studies had already been identified. The full text of the remaining 191 studies was reviewed and 66 studies [35-100] were considered relevant and included in the analysis in the final stage.

Additional file 2: Table S2 summarizes the effectiveness and cost-effectiveness of each HIV prevention intervention based on the reviews of domestic and international studies. It was not surprising that a much larger proportion of effectiveness and cost-effectiveness studies were conducted in international settings, mainly the US and Sub-Saharan Africa. In both the Thai and the international settings there were more effectiveness studies than cost-effectiveness ones (11 and 48 effectiveness studies in the Thai and international settings, respectively, versus 3 and 18 cost-effectiveness studies in each of the respective settings).

Most of the assessments focused on interventions affecting knowledge, attitudes and beliefs (36/63 or 57%), followed by biological/biomedical interventions (16/63 or 25%), harm reduction interventions (9/63 or 15%) and, lastly, mitigation of barriers to prevention and negative social outcomes of HIV infection (2/63 or 3%).

### Effectiveness and cost-effectiveness of HIV prevention intervention

The findings demonstrated that male condoms use, street outreach programs, programs for the prevention of mother-to-child HIV transmission, circumcision, and needle and syringe programs were the only interventions to show strong evidence of reducing HIV infection among target populations.

**Figure 4:**
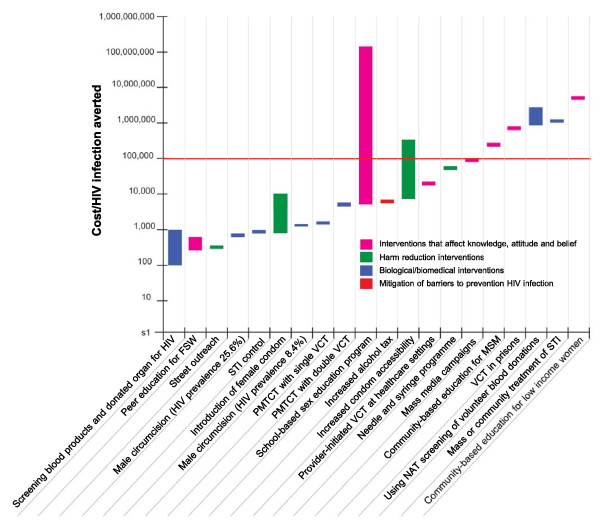
**Summary of cost-effectiveness data for HIV prevention intervention by type of interventions (PPP$ 2008 per HIV infection averted)**.

Figure 4 compares the cost per HIV infection averted of each HIV prevention intervention. It can be seen that the cost-effectiveness ratios vary largely, ranging from 70 PPP$ per HIV infection averted for screening blood product to 2,000,000 PPP$ per HIV infection averted for community-based education for low income women. It is likely that biological/biomedical interventions (highlighted in blue) are more cost-effective than those interventions affecting knowledge, attitudes and beliefs (highlighted in pink).

**Figure 5:**
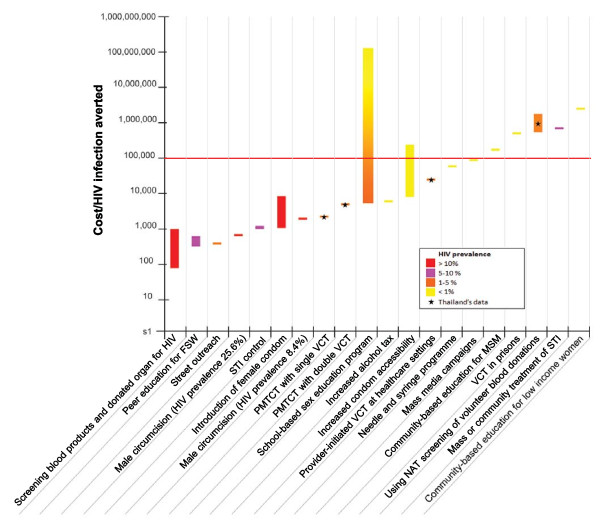
**Summary of cost-effectiveness data for HIV prevention intervention by type of interventions and HIV prevalence (PPP$ 2008 per HIV infection averted)**.

Figure 5 presents the modification of the figure 4 according to levels of HIV prevalence in settings where economic evaluations were conducted. It indicates that interventions performing in high HIV prevalence settings (e.g. screening blood products and donated organ for HIV or school-based sex education program in settings with high HIV epidemic) are likely to be cost-effective whereas interventions performing in low HIV prevalence settings (e.g. school-based sex education program in settings with low HIV prevalence or community-based education for low income women) are less likely to be cost-effective.

**Figure 6:**
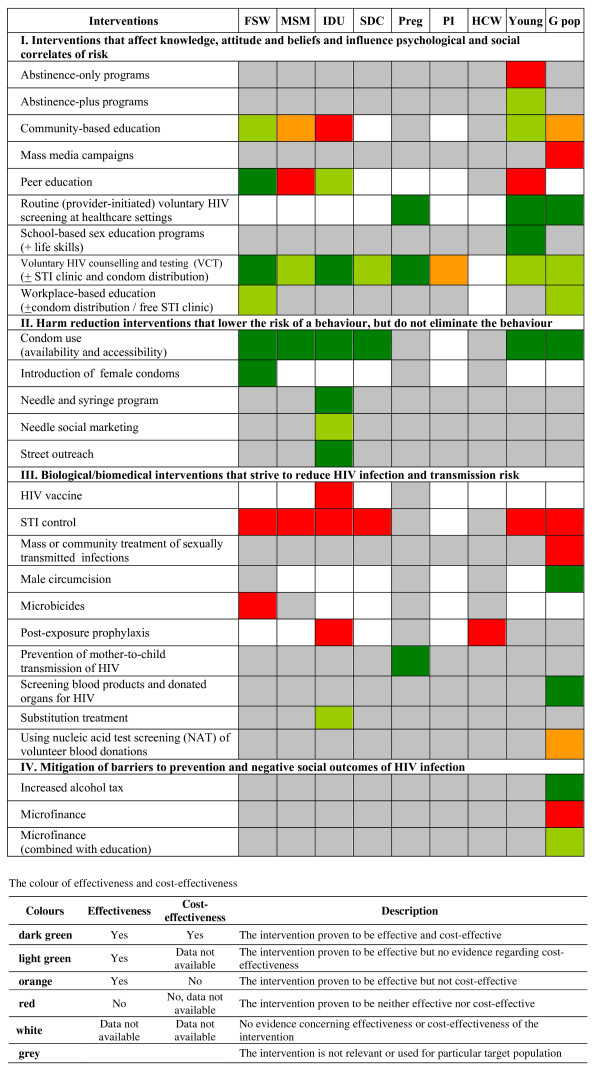
**Summary of findings by intervention and targeted population.** Abbreviations FSW - female sex worker; MSM - men who have sex with men; IDU - injecting drug user; SDC - serodiscordant couples; Preg - pregnant women; PI - prison inmate; HCW - healthcare worker; Young - people aged 10-24 years old; G pop - general people.

Figure 6 summarises the findings from the review and prioritises HIV prevention interventions based on effectiveness and cost-effectiveness evidence. The table presents results by targeted population including female sex workers, IDUs, men who have sex with men and serodiscordant couples, who are currently the major sources of HIV infection in Thailand.

Those interventions proven to be both effective and cost-effective for female sex workers were: voluntary HIV counselling and testing, peer education, and male and female condom use. Community-based education and workplace-based education proved to be effective, but no evidence regarding the value for money among female sex workers was found. Although the use of microbicides is still in the trial phase, the evidence so far suggests that microbicides and sexually transmitted infections (STI) control were not effective in preventing HIV transmission amongst female sex workers.

Condom use was proven to be the only effective and cost-effective intervention for men who have sex with men while voluntary HIV counselling and testing demonstrated effectiveness but lacked cost-effectiveness information. Community-based education was clinically effective but cost-ineffective. Peer education and STI control were shown to be ineffective amongst this population.

For injecting drug users, voluntary HIV counselling and testing, condom use, needle and syringe programs, and street outreach were amongst the programs shown to be both effective and cost-effective. Needle social marketing, peer education, and substitution treatment demonstrated clinical effectiveness but were unsupported by economic evidence. Community-based education, HIV vaccines, STI control and post-exposure prophylaxis were shown to be ineffective in the prevention of HIV transmission amongst IDUs.

Condom use was the only interventions proven to be both effective and cost-effective for serodiscordant couples. Voluntary HIV counselling and testing was proven to be clinically effective but no cost-effectiveness information was available. STI control was not effective in preventing HIV transmission amongst this population.

With respect to information availability, voluntary HIV counselling and testing and condom use were the only interventions with extensive evaluations concerning effectiveness and cost-effectiveness across the different population groups. The main identified information gaps were for the following interventions: routine (provider-initiated) voluntary HIV screening at healthcare settings, introduction of female condoms, HIV vaccine, male circumcision, microbicides, and post-exposure prophylaxis. The evidence was particularly scarce for some of the targeted populations, namely serodiscordant couples, prison inmates and health care workers.

## Discussion

This review demonstrated several barriers to the use of effectiveness and cost-effectiveness evidence for policy decision making or program reorientation regarding HIV/AIDS. First, a lack of proper assessment about the effectiveness and/or cost-effectiveness outcomes of many interventions poses a significant challenge in making evidence-based health policy decisions. We found that most domestic studies evaluated the effectiveness or cost-effectiveness of interventions using only immediate measures such as knowledge, attitudes, perception, and skills. The use of such immediate measures severely limits the usefulness of the evaluations because they do not allow for the comparison of value for money across different types of interventions, due to variation in outcome measurement. In addition, these immediate outcomes may not be of primary interest to decision makers or health care planners when they consider health resource allocation.

Second, although high quality evidence was available for assessing many of the interventions' effectiveness, a major concern is the strength of evidence used to generate cost-effectiveness information. For example, many cost-effectiveness studies obtained the their effectiveness data from more bias-prone sources, such as expert opinion; in the case of the economic evaluation of HIV vaccine particularly unconvincing assumptions were applied [99]. This problem is also found in the economic appraisals of STI control and mass media campaigns which showed inconclusive clinical effects but good value for money [98,100]. Economic evaluation can be useful for guiding policy decisions only when it is performed correctly and reported accurately; these findings clearly depict barriers that would diminish the use of cost-effectiveness evidence to inform policy decisions.

Third, despite the emphasis we placed on finding local information on HIV prevention, the majority of studies identified in our reviews originate in other settings. Although their transferability/generalizability to the Thai settings might be questionable, it remains necessary to rely on these to inform policy making. In addition, most studies reporting the effectiveness and cost-effectiveness of HIV interventions were identified from international publications rather than domestic journals or grey literature (see table 2). This reflects the fact that good quality studies conducted both in Thailand or elsewhere are likely to be published in international journals. Thus, it is sensible to recommend that the international databases are still major sources of information, and can be used to inform decision making about the effectiveness and cost-effectiveness of HIV prevention interventions.

**Table 2:** Review profile of domestic literature

Type of literature	Initial search	Review of full text	Final inclusion
Articles published in domestic journals	528	16	1
Articles published in international journals	111	11	5
Theses/dissertations	99	11	5
Research reports	24	3	2
Conference proceedings	170	1	1

**Total**	**932**	**42**	**14**

The findings in this study are mostly consistent with the international literature, and notably the DCP2 findings [9]; male condoms, street outreach programs, programs for the prevention of mother-to-child HIV transmission, and circumcision were the only interventions to show strong evidence of reducing HIV infection among target populations. The DCP2 also identified these interventions, with the exception of circumcision, in its recommendations for concentrated epidemic areas including East Asia and the Pacific region. The differences between the recommendations from DCP2 and our findings are as follows:

• Although it was recommended in DCP2, there was a lack of strong evidence to indicate that community-based education offers good value for money in the prevention of HIV infection in either low or high HIV prevalence settings.

• In our review there were very consistent results showing that screening blood products and donated organs for HIV is very cost-effective while there was little reference to this intervention in the DCP2.

• This study found that interventions that aim to mitigate barriers to prevention and negative social outcomes of HIV infection such as increased alcohol tax and micro-financing also held promise of being effective. These interventions should be assessed in the future.

The differences between the DCP2 report and this study may be explained by the fact that DCP2 did not employ a comprehensive and systematic search for evidence, resulting in a number of published and unpublished studies being excluded. Furthermore, this study gave a higher priority for evidence from Thai context at which the recommendations are primarily aimed, while the DCP2 report aims to provide policy recommendations across a broader range of health care settings.

It is interesting to note that we found very limited local information about HIV interventions amongst specific high risk populations (i.e. IDUs, men who have sex with men, female sex workers, and young people). Of the nine interventions conducted in Thailand identified in our review, only one study focused on a high risk group, assessing an HIV vaccine for injecting drug users. These findings underline the urgent need to prioritize health research resources to assess the effectiveness and cost-effectiveness of HIV interventions aimed at the reduction of HIV infection among high risk groups.

Caution should be exercised when comparing the effectiveness and cost-effectiveness data in this study to inform policy decision making. Firstly, because many studies were conducted in various settings with different sized target populations, different HIV prevalence, different attitudes towards HIV/AIDS, and varying socio-economic and cultural determinants of behavioral responses to interventions; these factors would greatly affect not only the effectiveness of the intervention but also its value for money.

Secondly, although we have made explicit criteria to judge whether the effectiveness studies/data are good enough to be used in decision making, there was no such standard to measure the quality of cost-effectiveness studies. We found that most of the effectiveness studies are of good quality (mainly in the 1st or 2nd hierarchy) but we are in doubt of the quality of data used in many of the cost-effectiveness studies.

Lastly, it is important to recognize that effectiveness and cost-effectiveness are not the only criteria in guiding health care rationing and that political and ethical dimensions and other societal values such as equity can also play significant roles in decision making processes. However, these issues were not under consideration in this study.

## Conclusion

This study found that school based-sex education plus life-skill programs, voluntary and routine HIV counselling and testing, male condoms, street outreach programs, needle and syringe programs, programs for the prevention of mother-to-child HIV transmission, male circumcision, screening blood products and donated organs for HIV, and increased alcohol tax showed strong evidence and value for money in reducing HIV infection among target populations.

We found very limited local evidence regarding the effectiveness of HIV interventions among high risk populations in Thailand. This underlines the urgent need to prioritise health research resources to assess the effectiveness and cost-effectiveness of HIV interventions aimed at reducing HIV infection among high risk groups.

This review also demonstrated several limitations in using effectiveness and cost-effectiveness evidence for policy decision making concerning HIV/AIDS. First, a lack of proper assessment about the effectiveness and/or cost-effectiveness outcomes of many interventions poses a significant challenge in making evidence-based health policy decisions and program reorientation. Second, although good quality evidence was available for assessing intervention effectiveness, a major concern is the strength of evidence used to generate the cost-effectiveness information. Third, although we put more effort into identifying local information on HIV prevention, a majority of the studies included in the final analysis were identified from international databases rather than local sources, and may not be entirely applicable in the Thai context.

## Competing interests

The authors declare that they have no competing interests.

## Authors' contributions

All named authors contributed jointly to the conception, data collection, analysis, and writing of the report. All authors read and approved the final manuscript.

## Pre-publication history

The pre-publication history for this paper can be accessed here:

http://www.biomedcentral.com/1471-2458/10/401/prepub

## Supplementary Material

Additional file 1 **Table S1 Classification and definition of HIV prevention interventions in the review**. The table describing the classification and definition of HIV prevention interventions in the review (11 pages). Click here for file

Additional file 2 **Table S2 Summary concerning the effectiveness and cost-effectiveness evidence of HIV prevention interventions**. The table describing the effectiveness and cost-effectiveness evidence of HIV prevention interventions (30 pages). Click here for file
