# Supplementary material for: Reviewing the evidence on effectiveness and cost-effectiveness of HIV prevention strategies in Thailand
Source: BMC Public Health. 2010 Jul 7;10:401. doi: 10.1186/1471-2458-10-401 (PMC2912810; doi:10.1186/1471-2458-10-401)
Supplement: Additional file 1 — Table S1 Classification and definition of HIV prevention interventions in the review. The table describing the classification and definition of HIV prevention interventions in the review (11 pages). [file 1471-2458-10-401-S1.PDF]

Table S1 Classification and definition of HIV prevention interventions in the review

Name of intervention	Activities, services, commodity	Message content (if relevant)	Delivery mode	Target population/ setting	Outcomes/ theory
I. Interventions affecting knowledge, attitudes and beliefs and influencing psychological and social risk correlates					
**Abstinence**	Abstinence-only programs often target family involvement and community norms, as well as individual behaviours by addressing multiple influences on knowledge, attitudes, and values. Abstinence-plus programs promote sexual abstinence as the best means of preventing HIV, but also encourage condom use and other safer-sex practices for sexually active participants.	The social, health-related, and psychological benefits of abstaining from sexual activity--most of them note the potential harm of sexual activity outside marriage	Varies	young people (10-24 years) who may not yet have initiated sexual activity	To encourage both primary abstinence (remaining a virgin) and secondary abstinence (returning to abstinence after sexual activity) to refrain from sexual activity. Theoretical underpinnings include social learning theory, the health-belief model, cognitive-behavioural theory, the theory of social inoculation, the culture of poverty perspective, and utility maximization perspectives
**Community-based education (including opinion leader programs)**	These programs affect community-wide behaviour change. In this approach, popular opinion leaders are trained to disseminate risk reduction messages to their peers, and thereby influence other group members to re-evaluate their	Varies	Varies	Broad population base	Social change theory

Name of intervention	Activities, services, commodity	Message content (if relevant)	Delivery mode	Target population/ setting	Outcomes/ theory
	own HIV risk, modify their attitudes toward safer sexual practices, and change their behaviour.				
**Peer education intervention**	The peer education intervention is a model of training that supports participants to develop and then deliver information to their peers.	Varies: e.g. mitigation of stigma and discrimination towards people living with HIV	Peer educators, trained outreach workers	Typically targeted to smaller, unique populations	Varies: includes diffusion-based interventions that strive to affect behaviour through the dynamics of social networks
**Life Skills-Based Education (LSBE)**	LSBE refers to an interactive process of teaching and learning which enables learners to acquire knowledge and to develop attitudes and skills which support the adoption of healthy behaviour.	It is being adopted as a means to empower young people in challenging situations.	Varies	Young people (10-24 years)	Enhanced self-efficacy
**Mass media campaigns**	Mass communication potentially to influence social norms, expectation and behaviour related to HIV/AIDS	Varies(e.g. people in the community are at risk of HIV infection through sexual behaviour)	Television, radio, public events	Typically large segments of the population, but content can be targeted to subpopulations	Varies: reduced HIV-related risk behaviour, changes in social norms
**Provider-initiated HIV**	All patients are offered HIV testing and consent to be tested is implied as with	-	Healthcare providers	People visiting hFacilities for any	To increase uptake of VCT and early recruit to ART if

Name of intervention	Activities, services, commodity	Message content (if relevant)	Delivery mode	Target population/ setting	Outcomes/ theory
**counselling and testing (PICT)**	any other clinically indicated laboratory test; patients may opt out if they do not want to be tested.			purpose	positive, or maintain low risk behaviour in the population when detected negative
**School-based education**	School-based education programs, an aspect of information, education, and communication, provide information to young people and reinforce healthy norms in a school setting.	Varies	Teacher, healthcare provider	School children	Varies
**Voluntary counselling (with/without HIV testing)**	Individuals or groups of people are taught about HIV/AIDS. When HIV testing is performed, counsellors encourage their clients to notify them of their HIV status and provide counselling support to help them cope with the outcome. This intervention must be performed on a voluntary basis.	Causes and risk factors of AIDS, the steps necessary to prevent HIV infection, and how to prevent the spread of the disease for those who have already been infected with HIV	Trained counsellor	Varies	Varies
**Workplace-based education (including**	These programs communicates AIDS prevention messages to employees in either formal or informal settings, provide a role model for behaviour	Varies	Healthcare provider, peer-educator, trainer	Employee	It induced changes in knowledge, attitudes, and risk behaviour.

Name of intervention	Activities, services, commodity	Message content (if relevant)	Delivery mode	Target population/ setting	Outcomes/ theory
**prison-based education)**	change, and distribute and demonstrate the correct use of condoms.				
II. harm reduction interventions that lower the risk of a behaviour, but do not eliminate the behaviour					
**Male and female condom use and/or distribution**	These programs provide free condoms in readily visible and accessible sites through health care facilities and private businesses (through social marketing) serving populations at high risk of STDs and HIV.	-	Varies, but typically free distribution in public settings	Sexually active at-risk individuals	Decrease risk from unprotected sexual intercourse
**Needle and syringe exchange**	These programs provide a way for those IDUs who continue to inject to safely dispose of used needles and syringes and to obtain drug injection equipment at no cost. It provides a range of related prevention and care services that are vital to helping IDUs reduce their risks of acquiring and transmitting blood-borne viruses as well as maintain and improve their overall health.	-	Most typically community-based	Injecting drug users	Decrease use of contaminated injection equipment
**Needle social marketing**	The intervention aimed to reach all IDUs at both detoxification centres and	-	Most typically community-	Injecting drug users	Decreased use of contaminated injection

Name of intervention	Activities, services, commodity	Message content (if relevant)	Delivery mode	Target population/ setting	Outcomes/ theory
	local health institutions e.g. drug stores, community hospitals and private clinics. In detoxification centres, the intervention mainly consisted of health education provided by health workers. In the community, health workers or peer educators visited drug users' homes or places where they gathered. The Intervention included face-to-face health education, dispensing and recalling needles. Drug users could also collect materials/needles from the local hospitals or centres for Disease Control (CDC) and from peer educators.		based		equipment
III. biological/biomedical interventions that strive to reduce HIV infection and transmission risk					
**Anti-retroviral prophylaxis for vertical HIV transmission**	A combination of HIV counselling and testing, anti-retroviral prophylaxis and breastfeeding substitution. The Thai PMTCT program provides free services for voluntary HIV counselling and testing (VCT) for all pregnant women (approximately 0.8 million per annum),	-	Primarily clinic-based, which is linked to antenatal services	Infants born to HIV-positive mothers	Reduction in mother-to-child transmission and prevalence/incidence of HIV positive infants

Name of intervention	Activities, services, commodity	Message content (if relevant)	Delivery mode	Target population/ setting	Outcomes/ theory
	at first antenatal visit and at 28 weeks. HIV infected pregnant women receive free antiretroviral drugs, breast milk substitutes for 12 months and counselling with their partner to test their newborn babies at 12 and 18 months, and recruit them into universal ART programs when CD4 counts indicate the necessity.				
**STI control**	The intervention consists of multiple synergistic activities e.g. training health workers regarding syndromic management of STIs, supply of drugs and supervision, introducing STIs treatment guidelines, providing sexual education, condom distribution, etc.	-	Healthcare provider and community-based	Varies	Reduced prevalence of sexually transmitted infections—thought to also reduce HIV incidence
**Drug treatment including drug substitution treatment**	Oral methadone is the pharmacological agent that is most commonly used for substitution treatment of opioid dependence worldwide. There are two types of interventions. 1) methadone maintenance treatment (60 mg/day or	-	Healthcare provider	Injecting drug users/specialist drug and alcohol treatment program	Decreased dependence on injecting drugs and therefore minimize use of contaminated injecting equipments

Name of intervention	Activities, services, commodity	Message content (if relevant)	Delivery mode	Target population/ setting	Outcomes/ theory
	more) 2) Detoxification, the schedule is completed in 90 days. Data about HIV risk behaviour was reported for weeks one and two of treatment while participants were stabilised on methodone (40 mg/day) and weeks five and six at the commencement of the dose taper				
**HIV vaccine**	The first efficacy trial (Phase III) in Thailand of an HIV candidate vaccine (containing gp120 B and E subtypes) was initiated in 1999. It was conducted among injection drug users attending 17 Bangkok Metropolitan Administration (BMA) drug-treatment clinics. Eligibility criteria were: aged 20-60 years, drug injection during the previous year, being negative for HIV-1 by ELISA at screening and baseline. Vaccine or placebo was injected intramuscularly at months 0, 1, 6, 12, 18, 24, and 36 (36 months of follow-up). The primary end point for	-	Healthcare provider	Varies	Reduced incidence of HIV infection

Name of intervention	Activities, services, commodity	Message content (if relevant)	Delivery mode	Target population/ setting	Outcomes/ theory
	vaccine efficacy was HIV-1 infection.				
**Male circumcision**	Male circumcision is the surgical removal of all or part of the foreskin of the penis.	-	Healthcare provider	Males/typically clinic-based	Reduced biological risk of HIV acquisition
**Mass or community treatment of sexually transmitted infections**	The treatment consisted of azithromycin (1,000 mg single dose oral), ciprofloxacin (250 mg single dose oral) and metronidazole (2·0 g oral). Ciprofloxacin (FDA category C) was not given to pregnant women, who instead received cefixime 400 mg oral. Metronidazole (2·0 g oral) is the recommended single-dose regimen for trichomoniasis and provides short-term remission in 70–85% of cases of bacterial vaginosis; it is safe in pregnancy (FDA category B). Benzathine benzylpenicillin (2·4 million IU intramuscular injection) was given in the home to TRUST (Toluidine Red Unheated Serum Test--the syphillis screening)-positive intervention-group	-	Healthcare provider	All consenting adults aged 15-59 years were given directly observed treatment of STI at home every ten months, irrespective of laboratory testing results or the presence of symptoms.	Reduced prevalence of sexually transmitted infections—thought to also reduce HIV incidence

Name of intervention	Activities, services, commodity	Message content (if relevant)	Delivery mode	Target population/ setting	Outcomes/ theory
	participants within 24 hr of serum collection; treatment was based on serological findings, since the administration of injections to uninfected individuals would be unacceptable. The drug regimen was given over 2 days (azithromycin and ciprofloxacin in day 1; metronidazole and intramuscular benzathine benzylpenicillin on day 2).				
**Microbicides**	Microbicides are compounds formulated as gels, films, foams, suppositories, or creams and which, when inserted into the vagina, will prevent male-to-female transmission of HIV and other STIs. Nonoxynol-9, one potential vaginal microbicide, is widely used spermicide. The dosage ranged from 70 to 1,000 mg depending on the dosage form.	-	Varies, but typically free distribution in public settings	All women were advised to use vaginal microbicides prior to each episode of intercourse.	One of the important concepts in vaginal microbicide development is that it is a female-controlled method that does not necessarily require negotiation with a male sexual partner for use especially in the context of lower power relationship.
**Post-exposure prophylaxis**	Two or more antiretroviral drugs are recommended for duration of 4 weeks	-	Healthcare provider	Healthcare workers, rape	Reduced incidence of HIV infection

Name of intervention	Activities, services, commodity	Message content (if relevant)	Delivery mode	Target population/ setting	Outcomes/ theory
	to reduce the likelihood of HIV infection after potential exposure, either occupationally or through sexual intercourse.			victims and others exposed to biohazardous material	
**Screening blood products and donated organ for HIV**	Blood screening should be anonymous, the test result cannot be linked with the person whose blood has been tested, other than by the person themselves or a counsellor. Normally the blood sample is given a number or code, so that the person can be contacted if their results are positive.	-	Healthcare provider	Recipients of blood products and donated organs	Reduction in iatrogenic transmission of HIV through transfusion of blood and blood products
IV. mitigation of barriers to prevention and negative social outcomes of HIV infection					
**Microfinance**	The intervention employs such assets as savings accounts, family microenterprises, and scholarships to fight poverty and promote health and social functioning. For example; loans were administered for the development	-	Varies:individuals, microfinance and microcredit, social protection,	Individuals and families economically affected by AIDS	Economic empowerment. May also reduce secondary transmission of HIV

Name of intervention	Activities, services, commodity	Message content (if relevant)	Delivery mode	Target population/ setting	Outcomes/ theory
	of income generating activities with a group lending model.		insurance		
**Increases in alcohol taxes**		-	Legal system	Legislators, politicians decision-makers	A more restrictive alcohol policy through supply and demand side interventions reduces alcohol consumption, which in turn decreases risky sexual activity.
