# Supplementary material for: Reviewing the evidence on effectiveness and cost-effectiveness of HIV prevention strategies in Thailand
Source: BMC Public Health. 2010 Jul 7;10:401. doi: 10.1186/1471-2458-10-401 (PMC2912810; doi:10.1186/1471-2458-10-401)
Supplement: Additional file 2 — Table S2 Summary concerning the effectiveness and cost-effectiveness evidence of HIV prevention interventions. The table describing the effectiveness and cost-effectiveness evidence of HIV prevention interventions (30 pages). [file 1471-2458-10-401-S2.PDF]

Table S2 Summary concerning the effectiveness and cost-effectiveness evidence of HIV prevention interventions

Interventions	Population	Effectiveness			Cost-effectiveness			
		Level of evidence	Settings	Findings	Perspective / Approach	Setting	Comparators	Incremental cost-effectiveness ratio(s)
**I. Interventions affecting knowledge, attitudes and beliefs and influencing psychological and social risk correlates**								
Abstinence-only programmes	Young people	A	High-income countries	No evidence that the programmes can reduce HIV risk [39].	NA	NA	NA	NA
Abstinence-plus programmes	Young people	A	High-income countries	It found a significantly protective effect on sexual risky behaviours i.e. incidence and frequency of unprotected/protected sex; number of sexual partners; increased condom use. However, no significant effect on biological outcomes i.e. incidence of STI and pregnancy [37, 38].	NA	NA	NA	NA
Community-based	Young girls	B	US	During 3-12 months of follow-up at a health care	NA	NA	NA	NA

Interventions	Population	Effectiveness			Cost-effectiveness			
		Level of evidence	Settings	Findings	Perspective / Approach	Setting	Comparators	Incremental cost-effectiveness ratio(s)
education				setting, the intervention reduced sexual risk behaviours (e.g. vaginal sex without use of condom, giving oral sex, and alcohol and drug use before sex) [35, 40].				
Community-based education	Young people in rural areas	B	South Africa	There was no significant improvement for HIV sero-status and sexual risk behaviours after 2 years follow-up [36].	NA	NA	NA	NA
Community-based education	Injecting drug users	D+	Thailand	Drug use and sharing injection equipment with others was not significantly decreased after 1 month follow-up [33].	NA	NA	NA	NA
Community-	Women	B	US	The intervention improved	Societal /	US	‘do nothing’	ICER is PPP$

Interventions	Population	Effectiveness			Cost-effectiveness			
		Level of evidence	Settings	Findings	Perspective / Approach	Setting	Comparators	Incremental cost-effectiveness ratio(s)
based education	living in low income housing developme-nts			HIV knowledge, partner communication, risk-reduction behavioural intentions, and condom use, and decreased perceived barriers to condom use after 6-12 months follow-up [42, 43].	model-based economic evaluation			2,551,240 per HIV infection averted [42].
Community-based intervention (Sonagachi)	Female sex worker	C	India	HIV prevalence among sex workers (< 10%) had been lower than the national average (~30%) [44].	NA	NA	NA	NA
Community-based education (including opinion leader programme)	Men who have sex with men	A	Various	The interventions were effective in reducing unprotected sex by 35% at follow-up intervals ranging from 4 months to 1 year. They were also effective in	Societal / model-based economic evaluation	US	‘do nothing’	ICER is PPP$ 165,346 per HIV infection averted [47].

Interventions	Population	Effectiveness			Cost-effectiveness			
		Level of evidence	Settings	Findings	Perspective / Approach	Setting	Comparators	Incremental cost-effectiveness ratio(s)
				increasing reported condom use during anal intercourse by 59 % [41, 46].				
Mass media campaigns	general population aged 17-45 years	C	Various	Inconclusive results of mass media campaigns in terms of changing HIV risk behaviours were observed [98]. In examination of outcomes across studies, there is no statistically significant on the impact of mass media campaign.	Health care provider's / model-based economic evaluation	US	'do nothing'	ICER is PPP$ 87,124 per HIV infection averted [45].
Peer education intervention	Injecting drug users	B	US	After 6 months of follow-up, the intervention produced a 29% greater decrease in overall injection risks relative to the control (OR 0.71; 95%CI 0.52- 0.97), and a	NA	NA	NA	NA

Interventions	Population	Effectiveness			Cost-effectiveness			
		Level of evidence	Settings	Findings	Perspective / Approach	Setting	Comparators	Incremental cost-effectiveness ratio(s)
				76% decrease compared with baseline. Sexual risk behaviours and safe injection were also decreased from baseline, but they did not differ between trial arms [53, 57].				
Peer education intervention	Female sex worker	C	Kenya	Peer-mediated interventions were associated with an increase in protected sex after 5 years follow-up. Female sex workers (FSW) who received peer interventions had more consistent condom use with clients compared with unexposed FSW (86.2% vs 64.0%; adjusted OR 3.6, 95%CI 2.1–6.1). These	Health care provider's / model-based economic evaluation	India/ Cameroon	'do nothing'	ICER of the mixed interventions targeted sex workers ranged from PPP$ 279 to 566 per HIV infection averted [49, 52].

Interventions	Population	Effectiveness			Cost-effectiveness			
		Level of evidence	Settings	Findings	Perspective / Approach	Setting	Comparators	Incremental cost-effectiveness ratio(s)
				differences were larger among FSW with greater peer-intervention exposure. HIV prevalence was 25% (17/69) in FSW attending ≥ 4 peer-education sessions, compared with 34% (25/73) in those attending 1–3 sessions (P=0.21) [54].				
Peer education intervention	Men who have sex with men	C	UK, Scotland	Peer education had less effective in sexual behaviour change among homosexual men. No significant different between control and interven-tion group in the proportion reporting unprotected anal intercourse (OR 1.12, 95%CI 0.81- 1.55) and negotiated	NA	NA	NA	NA

Interventions	Population	Effectiveness			Cost-effectiveness			
		Level of evidence	Settings	Findings	Perspective / Approach	Setting	Comparators	Incremental cost-effectiveness ratio(s)
				safety (OR 1.11, 95%CI 0.79-1.57) [50, 51, 58].				
Peer education intervention	Young people	C	Italy, US, Kenya	The intervention improved neither condom use nor number of sexual partners after 2 years follow-up. The percentage of students reporting condom use during the most recent sexual intercourse slightly decreased from 55.1% to 49.7% in intervention arm, though the decrease was not significant. The percentage of students with more than one partner was increased [48, 55, 56].	NA	NA	NA	NA
Routine (provider-	Adults aged 15-65	A+	Thailand	Routine provider offering of HIV screening significantly	Healthcare provider's /	Thailand	'no screening'	ICER is PPP$ 22,899.16 per HIV

Interventions	Population	Effectiveness			Cost-effectiveness			
		**Level of evidence**	**Settings**	**Findings**	**Perspective / Approach**	**Setting**	**Comparators**	**Incremental cost-effectiveness ratio(s)**
initiated) voluntary HIV screening at healthcare settings	years			increased the acceptance rate of HIV testing and the number of HIV infection detected compared to the standard practice of patient-initiated HIV testing (5.59% VS 0.32%) and (23 vs 10 HIV detection within 2 months in 8/8 case and control community hospitals), respectively [31].	economic evaluation alongside clinical study			infection averted [31].
School-based sex education programme (combined with life skills)	Young people	D+	Thailand	Three studies indicate the improvement of AIDS preventive behaviours i.e. decreased number of visits to night clubs, decreased incidence of watching arousal media, increased sporting	NA	NA	NA	NA

Interventions	Population	Effectiveness			Cost-effectiveness			
		Level of evidence	Settings	Findings	Perspective / Approach	Setting	Comparators	Incremental cost-effectiveness ratio(s)
				activities, decreased alcohol drinking, decreased number of sex partners, and increased rate of using condom in the experimental group [26, 27, 30]. Another study found that the sexual risk behaviour was significantly improved after 4 month follow-up [29].				
School-based sex education programme	Young people	B	US, Italy, Mexico	The results of meta-analysis of 12 controlled studies in the US indicated that the overall mean effect size for abstinent behaviour was very small (effect size=0.05, 95%CI 0.01-0.09) [60]. In addition, the intervention targeted to	Societal / model-based economic evaluation	India / US / Cameroon	'standard practice'	ICERs ranged from PPP$ 4,853 [61] to 137,950,790 [59, 63] per HIV infection averted.

Interventions	Population	Effectiveness			Cost-effectiveness			
		Level of evidence	Settings	Findings	Perspective / Approach	Setting	Comparators	Incremental cost-effectiveness ratio(s)
				improve sexual risk behaviour did not induce change in condom use or number of sexual partners after 1-year follow-up. The only apparent benefit was a greater improvement in knowledge of HIV [62].				
Voluntary HIV counselling and testing (VCT) at workplace	HIV-negative employee	B	Zimbabwe	Highly acceptable VCT did not reduce HIV incidence at 2-year follow-up. HIV incidence was higher in the intensive VCT arm (mean per-site HIV incidence 1.37 per 100 person-years follow-up (PYFU) than in the standard VCT arm (mean per-site HIV incidence 0.95 per	NA	NA	NA	NA

Interventions	Population	Effectiveness			Cost-effectiveness			
		**Level of evidence**	**Settings**	**Findings**	**Perspective / Approach**	**Setting**	**Comparators**	**Incremental cost-effectiveness ratio(s)**
				100 PYFU), but the difference was not significant (adjusted rate ratio 1.49; 95%CI 0.79-2.80) [67].				
Voluntary HIV counselling and testing (VCT) in Prisons	Prison inmates at or near their time of release	NA	NA	NA	Societal / model-based economic evaluation	US prisons	'no HIV counselling and testing provided at Prisons'	ICER of offering VCT at prisons was PPP$ 508,651 per HIV case averted [70].
Voluntary HIV counselling and testing (VCT) and STD services at both clinic setting and	Men who have sex with men	A	Various	The intervention delivered at the individual level was effective in reducing unprotected anal intercourse (UAI) by 43% OR 0.57, 95%CI 0.37–0.87). These effects were significant in both the short- (median 6	NA	NA	NA	NA

Interventions	Population	Effectiveness			Cost-effectiveness			
		Level of evidence	Settings	Findings	Perspective / Approach	Setting	Comparators	Incremental cost-effectiveness ratio(s)
community setting				months) and long-term (median 12 months). It also improves sexual risk behaviour: condom use with anal intercourse (OR 1.55, 95%CI 0.73–3.29), number of sex partners (OR 0.97, 95%CI 0.45–2.06), unprotected oral sex (OR 0.58, 95%CI 0.28 – 1.24), incident HIV (OR 0.62, 95%CI 0.36 –1.06) [46].				
Voluntary HIV counselling and testing (VCT) plus STI services and free	HIV sero-discordant couples	D	Zambia	The proportion of reported condom use increased from <3% to >80% and remained stable through > 12 months of follow-up. Since underreporting was common, HIV transmissions were still	NA	NA	NA	NA

Interventions	Population	Effectiveness			Cost-effectiveness			
		Level of evidence	Settings	Findings	Perspective / Approach	Setting	Comparators	Incremental cost-effectiveness ratio(s)
condom				detected when couples had reported always using condoms. DNA sequencing confirmed that 87% of new HIV infections were acquired from the spouse [64].				
Workplace-based education	Male conscripts in military camps	C+	Thailand	Intensive workplace-based education programme for male conscripts (that was applied for 15 months) has successfully decreased incidence of HIV infection by 50% during the period of two years but not statistically significant (RR 0.49, 95%CI 0.11-2.26) [21].	NA	NA	NA	NA
Workplace-based	Female sex workers	D+	Thailand	The risky sexual behaviour was significantly decreased in	NA	NA	NA	NA

Interventions	Population	Effectiveness			Cost-effectiveness			
		Level of evidence	Settings	Findings	Perspective / Approach	Setting	Comparators	Incremental cost-effectiveness ratio(s)
education ± condom distribution				the intervention group compared to the control group after 1 week follow-up [25, 34].				
Workplace-based education/condom distribution/ free STD clinic visits	Female sex workers	C	Indonesia, China	The intervention was effective for increasing condom use (from 55-60% to 67-85%, p<0.01) and reducing STD among sex workers at 12 months evaluation. The prevalence of gonorrhea fell from 26% to 4%, and chlamydia fell from about 41 to 26% [69, 72]. The prevalence of HIV remained low throughout the study [68].	NA	NA	NA	NA
II. harm reduction interventions that lower the risk of a behaviour, but do not eliminate the behaviour								

Interventions	Population	Effectiveness			Cost-effectiveness			
		Level of evidence	Settings	Findings	Perspective / Approach	Setting	Comparators	Incremental cost-effectiveness ratio(s)
'100% condom programme'	Male conscripts	D+	Thailand	The data suggests that increased condom use along with some decrease in the frequency of commercial sex among the military conscripts led to a marked decline in STI and also to a subsequent reduction in HIV incidence [22].	NA	NA	NA	NA
Condom use (availability and accessibility)	Sexually active heterosex-ual couples	C	Various (reviewed evidence)	The HIV incidence in the "always" condom user group was 1.14 (95%CI 0.56-2.04) per 100 person-years. The HIV incidence in "never" condom user group was 5.75 (95%CI 3.16-9.66) per 100 person-years. Overall effectiveness, the proportionate reduction in HIV seroconversion with	Healthcare provider's / model-based economic evaluation	US	'do nothing'	Increase availability /accessibility of condoms in low HIV prevalence population (1.6% in men and 0.6% in women) appears to be cost-effective with ICER ranged from PPP$ 7,669 to 247,775 per case of HIV averted [59, 66] or

Interventions	Population	Effectiveness			Cost-effectiveness			
		Level of evidence	Settings	Findings	Perspective / Approach	Setting	Comparators	Incremental cost-effectiveness ratio(s)
				condom use, was approximately 80% [71].				about PPP$ 22,065 per QALY saved [65].
Condom use and sex education	HIV serodiscor-dant couples	D+	Thailand, India, Uganda	Condom use with their regular partner reached 100% at one-month follow-up visit. At three-month follow-up, more than 90% of the participants reported having been able to communicate and felt more comfortable discussing AIDS with their partner, and very confident that they could refuse sex if their partner refused to use a condom (an increase from 70% at baseline, p=0.0001) [77].	NA	NA	NA	NA
Introduction of Female	Female sex workers	D	Kenya	The introduction of female condoms led to a small, but	No specify/ Health care	South Africa/	‘do nothing’	ICER ranged from PPP$ 934 to 7,863 per

Interventions	Population	Effectiveness			Cost-effectiveness			
		Level of evidence	Settings	Findings	Perspective / Approach	Setting	Comparators	Incremental cost-effectiveness ratio(s)
condom				significant, increase in consistent condom use with all partners. Adjusted odd ratio for consistent condom use after female condom introduction was 1.7 (95%CI 1.4 - 2.2) [81].	provider's	Kenya		HIV infection averted [49, 61].
Needle social marketing	Injecting drug users	B	China	Needle social marketing can reduce risky injecting behaviour and HIV transmission among injecting drug users after 12-month follow-up. Needle sharing behaviour dropped significantly from 68.4% to 35.3%. However, the number of needle-sharing partners and sharing water was	NA	NA	NA	NA

Interventions	Population	Effectiveness			Cost-effectiveness			
		Level of evidence	Settings	Findings	Perspective / Approach	Setting	Comparators	Incremental cost-effectiveness ratio(s)
				unchanged. The HIV infection rate decreased but was not statistically significant [83].				
Needle and syringe programme (under supervision of medical staff)	Injecting drug users	B	Canada	After 6 months of follow-up, it was found that more consistent use of a supervised safer injecting facility is associated with positive changes in injecting practices, including less reuse of syringes, increased use of sterile water, cleaning of injection sites and cooking/ filtering of drugs (OR 2 – 3, 95%CI 1.38 - 4.37) [80].	Societal / model-based economic evaluation	US	'do nothing'	ICER is PPP$ 53,285 per HIV infection averted [76].
Street outreach	Injecting drug users	C	Various	Injecting drug users changed their baseline drug-related	Health care provider's /	Ukraine	'do nothing'	ICER is PPP$ 309 per HIV infection averted

Interventions	Population	Effectiveness			Cost-effectiveness			
		**Level of evidence**	**Settings**	**Findings**	**Perspective / Approach**	**Setting**	**Comparators**	**Incremental cost-effectiveness ratio(s)**
				and sex-related risk behaviour. Significant reductions in drug injection, multi-person reuse of syringes and needles and other injection equipment was found. The studies also showed a significant growth in promoting entry into drug treatment and increasing needle disinfection. However, although there was a reduction among drug users concerning sex-related risks and an increase in condom use, the majority still practiced unsafe sex. Regarding dosage effects, the	model-based economic evaluation			[82].

Interventions	Population	Effectiveness			Cost-effectiveness			
		Level of evidence	Settings	Findings	Perspective / Approach	Setting	Comparators	Incremental cost-effectiveness ratio(s)
				longer the exposure to outreach-based interventions, the greater the reductions in drug injection frequency [73, 75]. At cross border areas between China and Vietnam, new injectors declined 3-14% after 36-month follow-up. HIV prevalence and estimated incidence fell by approximately half at the 24-month survey and by approximately three quarters at the 36-month survey in both areas (P<0.01) [74].				
III. Biological/biomedical interventions that strive to reduce HIV infection and transmission risk								
HIV vaccine	Injecting drug users	A+	Thailand	The phase III HIV vaccine trial in Thailand demonstrated	Not clearly specify /	Thailand	'do nothing'	At the assumption of 30% vaccine efficacy,

Interventions	Population	Effectiveness			Cost-effectiveness			
		**Level of evidence**	**Settings**	**Findings**	**Perspective / Approach**	**Setting**	**Comparators**	**Incremental cost-effectiveness ratio(s)**
				that the vaccines are safe and well tolerated. However, after 36-month follow-up, there was no difference in terms of new HIV infection between the vaccine and placebo arms (the vaccine efficacy was estimated at 0.1%, 95%CI -30.8% to 23.8%) [28].	model-based economic evaluation			the ICER of vaccination, HAART, and their combination were about PPP$ 265, PPP$ 2,158, and PPP$ 944 per DALY averted compared with the do-northing strategy [99].
STI control	General population or persons with suspected STI	A	Uganda, Zimbabwe	The interventions were insufficient to reduce HIV-1 incidence. During a median follow-up of 3-3.6 years, the incidence of HIV-1 did not differ between intervention and control groups (incidence rate ratio = 1.00, 95%CI 0.63-1.58 [78] and incidence rate	Healthcare provider's / economic evaluation alongside clinical study	Tanzania /US	'standard practice'	ICERs is PPP$ 916 per HIV infection averted [61].

Interventions	Population	Effectiveness			Cost-effectiveness			
		Level of evidence	Settings	Findings	Perspective / Approach	Setting	Comparators	Incremental cost-effectiveness ratio(s)
				ratio = 0.75, 95%CI 0.48-1.17 [100]).				
Male circumcision	Heterosex-ual male	B	Various (mainly Africa)	The results from the review of existing observational studies demonstrate a strong association between male circumcision and prevention of HIV, especially among high-risk groups [79, 93, 94]. Moreover, a randomised trial in Uganda showed that Male circumcision reduced HIV incidence in men without behavioural disinhibition after 24-month follow-up. HIV incidence was 0.66 cases per 100 person-years in the intervention group and 1.33	Health care provider's / model-based economic evaluation	South Africa/ US	'do nothing'	Male circumcision appears to be very cost-effective in areas with high HIV prevalence (PPP$ 1,668 per HIV infection averted in areas with HIV prevalence of 8.4% and PPP$ 548 per HIV infection averted in areas with HIV prevalence of 25.6%) [87]. However, this intervention is unlikely to be cost-effective in the US where baseline HIV prevalence is relatively

Interventions	Population	Effectiveness			Cost-effectiveness			
		Level of evidence	Settings	Findings	Perspective / Approach	Setting	Comparators	Incremental cost-effectiveness ratio(s)
				cases per 100 person-years in the control group (estimated efficacy of intervention 51%, 95%CI 16–72; p=0.006) [86].				lower (2%) and homosexual and infection from needle sharing were major causes of HIV infection [59, 79].
Mass or community treatment of STI	Adults aged 15-59 years	A	Rural areas in Uganda with high rates of HIV and STI	After three rounds of mass treatment (30 months) there was no evidence indicating that universal treatment of STI reduced new HIV infections (rate ratio of 0.97% with 95%CI 0.81 - 1.16) [92].	Healthcare provider's / model-based economic evaluation	Tanzania / US	'standard practice'	ICERs is PPP$ 694,605 per HIV infection averted [59].
Microbicides	Female sex workers	A	Various	There is no evidence that nonoxynol-9 protects against vaginal acquisition of HIV infection (RR 1.12, 95%CI 0.88-1.42). Nevertheless, the risk of genital lesions was	NA	NA	NA	NA

Interventions	Population	Effectiveness			Cost-effectiveness			
		Level of evidence	Settings	Findings	Perspective / Approach	Setting	Comparators	Incremental cost-effectiveness ratio(s)
				significantly greater among women receiving nonoxynol-9 (RR 1.18, 95%CI 1.02-1.36) [95].				
Post-exposure prophylaxis	Healthcare workers	C	Various (reviewed evidence)	No evidence suggests that offering post-exposure prophylaxis with Zidovudine lowers the rate of HIV infection compared to 'no intervention'. Please note that no studies were found that evaluated the effect of two or more antiretroviral drugs [97].	NA	NA	NA	NA
Post-exposure prophylaxis (using two antiretroviral	Men and women with a potential	D	US	There was not a significant difference in the proportions of sero-converters (85.7%) and non sero-converters	NA	NA	NA	NA

Interventions	Population	Effectiveness			Cost-effectiveness			
		Level of evidence	Settings	Findings	Perspective / Approach	Setting	Comparators	Incremental cost-effectiveness ratio(s)
drugs for 28 days and if subject reported having recently had a detectable plasma HIV RNA level, then a protease inhibitor was also offered.	sexual or injection drug use exposure to HIV in the previous 72 hours			(94.1%) who were initially prescribed antiretroviral drug (P=0.4) [91].				
Prevention of mother-to-child transmission of HIV	Pregnant women	A+	Thailand	A randomized clinical trial demonstrated that a combination of Zidovudine (AZT) and a single does of Nevirapine (NVP),	Healthcare provider's / model-based economic evaluation	Thailand	'do nothing'	Combining the administration of AZT and NVP is the most cost-effective drug option. Cost-

Interventions	Population	Effectiveness			Cost-effectiveness			
		Level of evidence	Settings	Findings	Perspective / Approach	Setting	Comparators	Incremental cost-effectiveness ratio(s)
				administered both to the mother during labour and to the newborn, is highly effective in prevention of HIV vertical transmission, resulting in only 2.2 (±0.6) % of children being born with HIV compared to 6.9 (±1.4)% in the AZT-only arm [23, 32].				effectiveness ratio per averted infection of single VCT (1D) is PPP$ 1,938. Cost-effectiveness ratio per averted infection of double VCT (2D) is PPP$ 4,412 [32].
Screening blood products and donated organs for HIV	Blood donations	NA	NA	NA	Healthcare provider's / model-based economic evaluation	US / Sub-Saharan Africa	'no test'	HIV antibody testing for donated blood is a cost-saving intervention in the US [84] and very cost-effective in Sub-Saharan Africa (ICER PPP$ 64-870 per HIV infection averted) [49, 61].

Interventions	Population	Effectiveness			Cost-effectiveness			
		**Level of evidence**	**Settings**	**Findings**	**Perspective / Approach**	**Setting**	**Comparators**	**Incremental cost-effectiveness ratio(s)**
Substitution treatment	Injecting drug users	A	Various	The follow-up interview ranged from one month to 18 to 24 months; it was found that the intervention was associated with statistically significant reductions in illicit opioid use, injecting use and sharing of injection equipment. It is also associated with reductions in multiple sex partners or exchanges of sex for drugs or money, but has little effect on condom use. The reporting period for assessment of HIV risk behaviour ranged from 2 weeks to 6 months, and it appears that the reductions in	NA	NA	NA	NA

Interventions	Population	Effectiveness			Cost-effectiveness			
		Level of evidence	Settings	Findings	Perspective / Approach	Setting	Comparators	Incremental cost-effectiveness ratio(s)
				risk behaviour relating to drug use does translate into reductions in cases of HIV infection [85, 88, 96].				
Using nucleic acid test screening (NAT) of volunteer blood donations	Blood donations	D+	Thailand	It was estimated that there were approximately 38 to 155 additional units of donated blood detected with hepatitis B and C and HIV compared to the current practice (serology screening without NAT) [24].	Healthcare provider's / model-based economic evaluation	Thailand	'serology test without NAT'	ICER of providing NAT for blood donations was PPP$ 100,923 – 404,498 per hepatitis B or C or HIV detection PPP$ 553,455 - 1,937,715 per HIV infection averted [24].
IV. Mitigation of barriers to prevention and negative social outcomes of HIV infection								
Increased alcohol tax	General population	NA	NA	NA	Health care provider's / model-based economic evaluation	US	'current practice'	ICER is PPP$ 5,484 per HIV infection averted [59].

Interventions	Population	Effectiveness			Cost-effectiveness			
		Level of evidence	Settings	Findings	Perspective / Approach	Setting	Comparators	Incremental cost-effectiveness ratio(s)
Microfinance	Community	B	Africa	The intervention did not affect HIV incidence (adjusted RR 1.06, 95%CI 0.66–1.69) or rate of unprotected sexual intercourse with a non-spousal partner (adjusted RR 0.89, 95%CI 0.66–1.19). The experience of intimate-partner violence was reduced by 55% (adjusted RR 0.45, 95%CI 0.23–0.91; adjusted risk difference –7.3%, 95%CI –16.2 to 1.5) [89].	NA	NA	NA	NA
Microfinance (combined with training intervention)	Female aged 14-35 year	D+	Africa	Young participants were likely to have protected sex at last intercourse with a non-spousal partner (adjusted risk ratio 0.76, 95%CI 0.60–0.96)	NA	NA	NA	NA

Interventions	Population	Effectiveness			Cost-effectiveness			
		Level of evidence	Settings	Findings	Perspective / Approach	Setting	Comparators	Incremental cost-effectiveness ratio(s)
				after 2 years follow-up when compared with controls. In addition, they had higher levels of HIV-related communication (adjusted risk ratio 1.46, 95%CI 1.01–2.12) and were more likely to have accessed voluntary counselling and testing (adjusted risk ratio 1.64, 95%CI 1.06–2.56) [90].				

CI - confidence interval, NA - not available, OR - odds ratio, QALY - quality-adjusted life year, RR - relative risk
